# Hypersensitivity to Lanolin: An Old–New Problem

**DOI:** 10.3390/life14121553

**Published:** 2024-11-26

**Authors:** Kinga Lis

**Affiliations:** Department of Allergology, Clinical Immunology and Internal Medicine, Ludwik Rydygier Collegium Medicum in Bydgoszcz, Nicolaus Copernicus University in Torun, Ul. Ujejskiego 75, 85-168 Bydgoszcz, Poland; kinga.lis@cm.umk.pl

**Keywords:** lanolin, hypersensitivity, lanolin paradox, dermatology

## Abstract

Lanolin is a fatty substance derived from sheep’s fleece. The ancient Greeks used the moisturizing and skin-protective properties of this substance. The technique of industrial production of lanolin was developed in Germany in the 19th century. Since then, this natural wax has become an extremely popular base for many different cosmetic and pharmaceutical preparations intended for the treatment and care of the skin. In addition to its medicinal and cosmetic applications, lanolin is also widely used for industrial purposes. Hypersensitivity to lanolin has raised many questions and controversies for almost 100 years. Although lanolin has significant dermoprotective properties and when applied to intact skin without inflammatory changes, it lubricates it, improves its lipid barrier, and maintains proper moisture, it can also cause contact hypersensitivity when in contact with pathologically changed or damaged skin. It can, in the same person, both protect and damage the skin, depending on the condition of the skin to which the cosmetic or medicine containing lanolin is applied. The nature of the observed reactions and the circumstances of their occurrence, as well as the lack of a clear answer to the question of whether this wax causes allergies or not, make this phenomenon one of the so-called dermatological paradoxes. Although unusual reactions to lanolin have been the subject of research for many years, they still raise many questions to which there is still no clear answer. This is mainly due to the imperfection and incompleteness of the available publications. Although many different studies have been published on hypersensitivity to lanolin, most of them are retrospective analyses of the results of routinely performed epidermal patch tests or descriptions of clinical cases. Such reports and analyses, although undoubtedly very important, are a poor tool for assessing the sensitizing potential of lanolin and/or its derivatives. It is difficult to determine the causative factors, to define lanolin allergens, to investigate immunological mechanisms, or to assess the clinical significance of this phenomenon. There is a definite lack of standardized studies on the nature of lanolin hypersensitivity involving well-selected groups of patients and healthy volunteers, which would be conducted in a reproducible manner under laboratory and/or clinical conditions. As of today, lanolin hypersensitivity seems to be both an old and new problem that still remains unresolved.

## 1. Introduction

Lanolin is a fatty substance, secreted by a sheep’s skin glands, which protects the animal’s fleece from moisture. It is generally believed that the ancient Greeks were the first to obtain raw wool fat from sheep’s wool and begin using it as a skincare cosmetic. The first writings presenting the process of boiling wool in water to extract the outer layer of fat come from Ancient Greece and date back to 700 BC. In 60 AD, the Greek physician Dioscorides developed an efficient method of obtaining wool fat from sheep fleece [1], and the product obtained as a result of this process, the so-called “oesypum”, is often mentioned in Greek medical writings dating from that period and in much later pharmacopoeias from various countries [2].

In 1882, Otton Braun and Oscar Liebreice developed a method of centrifuging wool fat from the water that remained after washing wool. This technique enabled the industrial production of huge amounts of lanolin. The name “lanolin” was proposed by Otto Braun. This term was created from the Latin words: “lano”—wool and “oleum”—oil, which literally meant “wool oil” and perfectly reflected the origin and nature of this substance [3]. Initially, the name “lanolin” was a patented trademark. Later, however, it began to be commonly used as the generic name of this substance.

**Figure 1:**
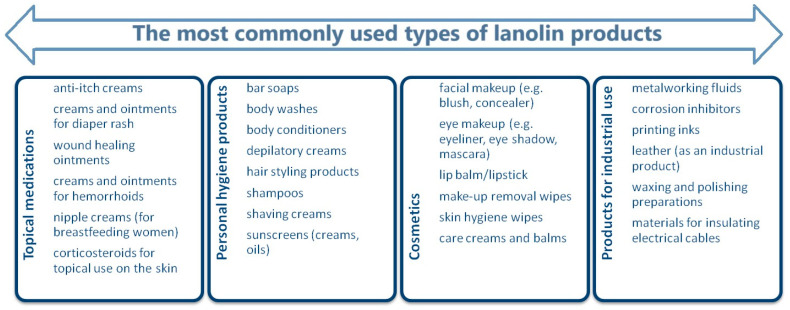
Commonly used types of products containing lanolin [4].

For the next 60 years, lanolin, due to its emollient and moisturizing properties, was one of the most common ingredients in both pharmaceutical and cosmetic skincare products. It is also the most frequently used base substance for various types of medicinal ointments (Figure 1) [4].

The first description of a case of contact allergy to lanolin, which occurred in a German patient in the form of a “skin reaction” to Nivea^®^ cream containing lanolin alcohol (eucerite), was reported in 1922 [5]. This description drew attention to a safety issue when using lanolin-based preparations in the context of the possibility of causing contact hypersensitivity. Since then, many case reports of lanolin allergy have been published and many retrospective analyses of this problem have been presented, which will be discussed later in this review. However, there is a lack of independent, well-designed studies on the allergenic properties of lanolin components. The existing studies were performed using well-characterized lanolin derivatives (free from impurities or with known impurities), conducted under standardized conditions, and involving a large group of clinically well-characterized patients and healthy volunteers. The only currently available studies of this type date back to the 1950s [6,7]. However, they were conducted with the participation of only a few patients who were randomly selected (the only condition for inclusion was a hypersensitivity reaction to lanolin products) and have never been repeated.

The problem of hypersensitivity to lanolin, although not new, still remains unresolved. The available retrospective analyses, based mainly on an evaluation of the results of routinely performed diagnostic tests, do not allow for unambiguous and objective conclusions about the nature of hypersensitivity reactions to lanolin derivatives. In addition, they encourage the creation of various assumptions or presumptions that are difficult to translate into clinical situations.

This review is an attempt to collect and summarize the currently available clinical case reports, retrospective analyses, and other publications on hypersensitivity to lanolin in order to draw attention to the simultaneously old and new, and still insufficiently investigated problem of hypersensitivity to lanolin and its derivatives.

## 2. Methodological Strategy of the Review

Publications reported in the PubMed and Google Scholar databases were analyzed. The search terms used and the preliminary results are presented in Table 1. The last verification of the available data was performed on 17 November 2024. A targeted search was conducted using generally available web browsers for specific publications cited in the analyzed studies, unless they were indicated in PubMed or Google Scholar. After analyzing the available data, the final compilation presents publications (case reports, retrospective analyses, and original studies) from the years 1950–2024.

## 3. Lanolin

Lanolin (wool wax, wool fat, wool tallow) is a purified secretory product of the sebaceous glands that is deposited on the wool fibers of sheep (*Ovis* spp.). Wool wax is obtained from sheep wool by washing. Raw lanolin constitutes approximately 5–25% of the weight of freshly sheared wool. The wool from one sheep can yield approximately 250–300 mL of recoverable wool fat [8,9].

Lanolin is a yellow, fatty substance with a yellow color (from light to dark), quite a hard consistency (density is approx. 0.9 g/cm^3^), and a specific smell. The melting point of lanolin is 36–42 °C. Heating the wool wax leads to its separation and the eradication of water. Lanolin is highly soluble in fats, sparingly soluble in cold alcohol (performing better in hot alcohol), and is easily soluble in benzene, chloroform, ether, carbon disulfide, acetone, and petroleum ether. Wool tallow does not dissolve in water, but instead absorbs water, creating an emulsion. This natural emulsifier can absorb a mass of water that is equal to twice its own weight, forming stable water/oil emulsions [9,10]. Lanolin is a very chemically unstable substance. It is sensitive to oxidation, which means that it is not stable in aerobic conditions (i.e., the usual conditions for the storage of cosmetics and medicines). The stability of lanolin in cosmetic and pharmaceutical products can be increased by the addition of antioxidants [11,12].

Lanolin is a substance of natural origin, which means that the share of individual substances and their mutual proportions vary, depending on the source material. It is a complex mixture of esters and polyesters. Lanolin does not contain triglycerides, so it is not a fat. The composition of this substance is typical of waxes and varies depending on its state of matter. The main components of lanolin are cholesterol, isocholesterol, and unsaturated monohydric alcohols, both free and combined with lanoceric acid, lanopalmitic acid, carnaubic acid, and other fatty acids. Lanolin also contains the esters of oleic and myristic acids and aliphatic alcohols such as cetyl, ceryl and carnaubyl alcohols, lanosterol, and agnosterol. Using solvent fractionation or crystallization, raw lanolin can be separated into lanolin wax (solid lanolin) and lanolin oil (liquid lanolin) (Table 2) [11,12,13].

The physical state of lanolin also affects its properties and suitability for specific applications (Table 3).

Lanolin, when used in cosmetic and pharmaceutical applications, is classified as an anhydrous lipophilic base. It has the ability to bind with water-soluble substances, which facilitates the absorption of active ingredients through the skin. Free sterols, their esters, and free and esterified fatty acids make lanolin highly effective for moisturizing [15,16,17].

The composition and structure of lanolin mimic the lipid matrix of the stratum corneum. Due to this similarity, lanolin components can penetrate the structures of the stratum corneum and react with corneocytes at the border of the granular and stratum corneum [18,19]. This allows for overcoming the natural barrier of the stratum corneum for hydrophilic substances and delivering various nutritional and/or therapeutic substances to the dermis [15,16].

Lanolin also has softening and occlusive properties and a natural ability to slow down the loss of moisture from the skin [20].

### 3.1. Forms, Fractions, and Derivatives of Lanolin

Lanolin wax and lanolin oil (Table 3) are the two basic fractions of lanolin. Other modified and unmodified forms of wool fat are also widely used. These substances have different compositions, which determine their physical and chemical properties. They can also penetrate the epidermis and affect the tissues with varying intensity and effectiveness. These individual characteristics of particular varieties and/or derivatives of lanolin predispose them to specific applications. The largest group of lanolin-derived substances are forms that are chemically modified by hydrolysis, hydrogenation, alkoxylation, acetylation, and transesterification [8,21].

#### 3.1.1. Anhydrous Lanolin

This is a yellowish, persistent, semi-solid fat with a slight odor. It is practically insoluble in water. It is sparingly soluble in alcohol and is easily soluble in benzene, acetone, ether, and petroleum ether. Anhydrous lanolin is able to absorb about twice as much water as it weighs. The melting point of anhydrous lanolin is about 40 °C [11,21]. Anhydrous lanolin is the final product in the lanolin extraction process and is used as a raw material with a wide range of applications in the pharmaceutical and cosmetic industries [11,12].

#### 3.1.2. Hydrated Lanolin

This form of lanolin contains 25–28% water (usually a ratio of 25% water to 75% fat). It is most often used for the production of lubricants and in wood preservation and fabric processing [11,12].

#### 3.1.3. Refined Lanolin

This is a pale yellow solid that is insoluble in water (although soluble in ether and chloroform). It contains 25–30% of absorbed water. A significant part of this form of lanolin consists of high-molecular-weight fatty acids and alcohols. Refined lanolin melts at 40 °C [11,12,21].

#### 3.1.4. Lanolin Alcohols

Lanolin alcohols, including modified lanolin alcohols (e.g., acetylated and oxyoxygenated alcohols and oxypropylene), have a much greater emulsifying ability than unmodified lanolin. For this reason, they are used as plasticizers and dispersants [8,11,12,21].

#### 3.1.5. Eucerite

Eucerite is a mixture of steroidal alcohols: cholesterol and isocholesterol, lanosterol, and higher aliphatic alcohols, obtained from the hydrolysesters contained in lanolin. The history of eucerite dates back to the early 20th century, when in 1902, the German chemist Isaac Lifschütz patented an emulsifying agent that he called “eucerite”, which means “beautiful wax” in Greek [22]. Eucerite became the basis for the development of the first-ever stable oil-in-water emulsions. Mixed with Vaseline, it produces Eucerin (or eucerol), and, in this form, it is the technological base for many medicinal and cosmetic creams, including the extremely popular NIVEA^®^ cream, which has been constantly present on the cosmetics market since 1911. Interestingly, the name of the cream “NIVEA” is derived from the Latin words “nix, nivis” (snow), which refer to the snow-white color of the cream [22].

Eucerin is highly soluble in water, so it is also used as a lubricating ingredient in syndetics, emollients, shampoos, tonics, varnishes, and nail polish removers, as well as in lipsticks and lip glosses [11,12,21,23].

#### 3.1.6. Lanolinic Acids

Lanolinic acids are a mixture of branched fatty acids, hydroxy acids, and other compounds with a low degree of saturation. This group of lanolin derivatives is obtained during the hydrolysis process. Lanolin acids are used as starting compounds for the synthesis of further lanolin derivatives, such as isopropyl esters, glycerol esters, and oxyethylene derivatives. Lanolin acids and their derivatives are used as solubilizers, emulsifiers, dispersants, and wetting agents in cosmetics and pharmaceuticals. Lanolin fatty acids have softening, lubricating, and shiny properties. They work very well in haircare cosmetics (shampoos, conditioners, and lotions) [11,12,21].

#### 3.1.7. Lanolin Esters

The isopropyl esters of lanolin acids are obtained by the esterification of lanolin fatty acids with isopropyl alcohol or the transesterification of raw lanolin. Lanolin esters are less viscous than lanolin and spread better on the skin [21]. Due to the presence of hydroxyl groups, they have surface-active properties, including wetting ability and dispersing pigments. The consistency of lanolin esters depends on their molecular weight and viscosity. Esters with low viscosity and low molecular weight are liquid in nature. The average molecular weight causes them to become substances with a soft and buttery consistency. High-molecular-weight esters are semi-solid [21,24].

The consistency of lanolin isopropyl esters determines their usefulness and application. Liquid ester derivatives are waterproof and are easily absorbed by human skin, strongly smoothing it. They are usually used in oil preparations for baby care. Derivatives of medium molecular weight, which have a buttery consistency, easily become liquid when applied to the skin. They are mainly used in toilet soaps, cosmetic creams, and lotions. Higher-molecular-weight semi-solid derivatives are non-greasy and have good emollient and lubricating properties. They are widely used in lipsticks to reduce drag and improve shine [21,24].

Interestingly, although lanolin isopropyl esters are the most widely used, it is also possible to esterify lanolin acids with alcohols other than isopropanol. For example, after esterification with glycerin, lanolin acids create a mixture of mono-, di-, and tri-ester glycerides, dominated by monoesters. They are typically used as emulsifying emollients in light creams and lotions. The glycerin esters of lanolin often leave a heavy, hydrophobic, non-greasy film on the skin, which can be easily washed off with water and detergents due to its poor resistance to these agents [21,24].

#### 3.1.8. Acetylated Lanolin Derivatives

Acetylated lanolin is an almost odorless, semi-solid, yellowish gooey substance. It has a lower melting point (36 °C) and reduced emulsifying properties than the starting substance. It is also more hydrophobic than lanolin. It dissolves in mineral oils and some vegetable oils. Acetylated lanolin disperses easily in an oil-in-water emulsion. It does not form a water-in-oil emulsion and is used as an auxiliary agent in pharmaceutical preparations and cosmetics, especially the group of sunscreen preparations especially intended for children [20,21,25].

#### 3.1.9. Amerchol L-101

Amerchol L-101 is a substance obtained from the hydrolysis of lanolin (lanolin alcohol or sheep wool alcohol). In cosmetics, Amerchol L-101 is used as a moisturizer and emollient. It is also a component of the base used in topical medicines. Amerchol L-101 is also used in industrial preparations, such as pastes for the care of furniture and leather products. It is also an ingredient of anti-corrosion preparations, paints, and inks. Amerchol L-101 is present in paper, textiles, furs, machine oils and waxes. Amerchol L-101 is also important as a substance that is commonly used in the diagnosis of a contact allergy to lanolin. The substance used for epidermal patch tests is Amerchol L-101 (10% lanolin alcohol dissolved in mineral oil) at 50% in Vaseline [4,12,26,27].

## 4. Allergy and/or Hypersensitivity to Lanolin

Interest in the problem of possible allergy to lanolin began at the turn of the 1920s and 1930s. In 1922, A. Marcus [5] presented a case concerning a skin reaction that occurred as a result of the use of Nivea^®^ cream containing Eucerin, which is considered the first description of hypersensitivity to lanolin. In 1931, Sulzberger and Morse [28] reported two cases of probable lanolin allergy. A description of three more cases of this hypersensitivity was provided by Sezary [29] in 1936. In turn, in 1939, Bonnevie [30], in a study of 2358 patients, identified a single case of lanolin allergy. In 1947, Ellis [31] described nine cases of contact allergic dermatitis caused by an ointment (“Aquaphor”) based on oxycholesterol, petroleum jelly, and lanolin-derived substances. The author tried to draw attention to the underestimated problem of hypersensitivity to medicinal substances applied to the skin, which may be caused by additional substances that constitute the basis for the preparation of the medicine.

Overall, until 1950, reported clinical cases of the various skin reactions associated with the use of cosmetic or medicinal preparations based on lanolin were rather few [32]. Due to the reports being so few, it was concluded that the problem of hypersensitivity to lanolin and its derivatives was of no major clinical importance, estimating the incidence of this phenomenon in a quite wide range from 0.25% to 18.6% in the general population [32]. It is worth noting that according to data from 2022, among North American patients with dermatitis, the incidence of contact allergy to lanolin ranges from 1.2% to 6.9% of these people [33]. In the European population, this allergy affects approximately 0.4% of people [34].

In the 1950s, subsequent case reports of skin reactions to lanolin again focused attention on this topic. Analysis of the published clinical data on hypersensitivity to lanolin suggests that the problem of allergy to wool wax and its derivatives particularly affects certain groups of patients whose risk of hypersensitivity to lanolin is higher than in the general population (Table 4).

Based on the data collected in Table 4 and the extensive analysis in the “Safety Assessment of Lanolin-Derived Ingredients as Used in Cosmetics” prepared by the Expert Panel for Cosmetic Ingredient Safety [12], it appears that hypersensitivity to lanolin particularly affects several groups of patients (Table 5).

## 5. In Search of Lanolin Allergens

The beginning of research on the allergenic potential of lanolin dates back to the 1950s. In 1950, Sulzberger and Lazar [6] published the results of their research, in which they focused on an analysis of the immunogenic properties of lanolin components, both their allergenic properties and the possibility of promoting an autoimmune response. These researchers associated the possible autoimmune potential of lanolin with the similarity of the components in wool fat to components in the secretions of human sebaceous glands (sebum) [6]. During their research, as a result of their analysis of epidemiological data on hypersensitivity to lanolin, Sulzberger and Lazar [6] drew attention to an interesting phenomenon regarding the small number of reported cases of allergic reactions to wool fat in the general population, in the context of the common exposure of people to contact with lanolin and its derivatives.

In the research of Sulzberger and Lazar [6], what draws attention to the allergenic properties of lanolin and its derivatives is the authors’ well-thought-out concept and attention to detail. Unfortunately, and importantly, these studies, although conducted over 70 years ago, still remain the best-planned and most thoroughly executed research project on hypersensitivity to lanolin. For this reason, in this review, both the study project developed by Sulzberger and Lazar [6] and its results are described in detail, in the hope that it will inspire readers to undertake new, definitely needed, studies in this area.

Sulzberger and Lazar [6] enrolled four people in their study. The inclusion criteria were a clinical history of contact hypersensitivity in the skin (Table 6) and a positive reaction in a 48-hour lanolin patch test. To exclude the primary irritating effect of the tested substances, three randomly selected people (without skin contact reactions) were tested with the same substances as the patients.

The study participants underwent a series of patch tests (48 h) using 12 selected substances that were lanolin components, lanolin from various sources, and chemical substances used in the extraction process of lanolin components. The substances used in the experiment were found in pharmaceutical and cosmetic preparations (base ingredients) (Table 7).

In all patients, a clearly positive reaction was observed to each of the lanolin preparations (substances Nos. 7–9), a proprietary ointment containing lanolin alcohols (substance No. 2 and No. 6), a fraction of mixed lanolin alcohol, and a fraction of lanolin alcohol mixed with fatty acids (substance No. 4). None of the other tested substances caused skin reactions (Table 8) [6].

Based on the presented results (Table 8), Sulzberger and Lazar [6] concluded that the immunologically active factor (responsible allergen) was a specific component or components present in the mixture of wool fat alcohols that are not present in other fractions (e.g., fatty acids, cholesterol, and lanosterols). However, this substance has not been definitively identified. The authors also did not find confirmation of their hypothesis about the relationship between hypersensitivity to lanolin and the autoimmune response to sebum.

In subsequent years, Sulzberger et al. [7] extended the previous project by testing the allergenicity of various lanolin derivatives (acetylated derivatives and aliphatic alcohols) and other additional substances (olive oil and cetyl alcohol) found in cosmetics and/or medicines. Nineteen patients with contact-type hypersensitivity were recruited for the study. The results of this project confirmed previous observations indicating that aliphatic lanolin alcohols were the most likely lanolin allergen. At the same time, these researchers noticed that acetylated lanolin derivatives are probably hypoallergenic [7]. Both of these observations were confirmed in the study by Everall and Truter [97], which showed that lanolin alcohols have allergenic properties and acetylation deprives them of this property. In turn, Clark et al. [98,99] noticed that removing the free fatty alcohol fraction from lanolin to a level below 3% results in a reduction in the incidence of hypersensitivity to lanolin by 96%–99%. Independent confirmation of the allergenic potential of lanolin alcohols was provided by the results of studies on an animal model [100,101,102]. Bourrinet and Berkovic [100,101] performed a series of patch tests with lanolin (fresh, in long-term storage, and purified), lanolin alcohols, and semi-liquid lanolin on the skins of guinea pigs. They observed positive reactions only for wool alcohols.

Another candidate for the allergenic component of lanolin was the lanolin sterol fraction [103], although it raised considerable doubts [104,105]. Fregert et al. [102] performed tests with various lanolin fractions on the skins of guinea pigs (including lanolin alcohols and sterol-like fractions) and observed that lanolin alcohols caused hypersensitivity reactions in 10 to 30% of animals, but two sterol-like fractions did not cause such reactions.

It is also worth considering another theory of lanolin allergenicity, which assumes that the long-term storage of wool fat may lead to the oxidation of lanolin and its derivatives. Oxidation changes the conformation of the molecules, which may increase their immunogenicity. Attention was drawn to the connection between the aging process of lanolin and the answer to the question of why some samples of lanolin and its derivatives sometimes cause hypersensitivity reactions and sometimes do not (in the same people) [33,106]. Hjorth and Trolle-Lassen [41] studied 20 patients who were initially diagnosed as sensitive to lanolin and performed patch tests with “fresh” Eucerin and “old” Eucerin (it was stored for 7 years in a tightly sealed package). These researchers noticed that all patients reacted to “old” Eucerin but only 55% of them also had positive results with “fresh” Eucerin. These results suggest that lanolin oxidation products may take on allergenic properties that they did not have before the oxidation process. The use of Eucerin and not lanolin is the weak point of this study. Its composition may vary depending on the origin, source material, and production series. This means that it is impossible to fully predict how the aging of this product will affect its properties because this depends on the share of individual fractions in a specific series of lanolin. For example, Bourrinet and Berkovic [101], when conducting tests on guinea pigs with samples of fresh and old lanolin, did not notice any difference in their sensitizing potential. Of course, the involvement of aging processes in modulating the allergenicity of lanolin cannot be completely ruled out, but this hypothesis certainly requires further, more advanced research with a larger group of patients.

The allergenic properties of lanolin may also be related to the natural origin of this substance. Products from natural sources may contain admixtures of various foreign substances that were present in the original sources. These foreign additives may cause hypersensitivity, regardless of the main substance (in this case, lanolin). It has been proven that sheep wool may contain pesticides from grass spraying, insecticides used to combat the external parasites of sheep, and residues of the detergents or other chemicals used for the washing and technological processing of wool to produce lanolin [99,107,108,109,110,111]. Even if their concentrations are not toxic [109], it cannot be ruled out that each of these “foreign substances” may cause allergies unconnected to lanolin.

## 6. The Unusual Face of Hypersensitivity to Lanolin—The ”Lanolin Paradox”

In the literature on hypersensitivity to lanolin, non-standard terms have also been used in an attempt to illustrate the unusual features of this allergy and the associated risk, such as the “lanolin paradox” [112], “lanolin myth” [105] or “lanolin—the wolf in the sheep’s clothing” [4]. All these terms are justified by the unusual phenomena and facts related to hypersensitivity to lanolin. If we take into account the general population’s exposure to the lanolin present in medicines, cosmetics, and many other industrial products and household chemicals, the percentage of people allergic to this substance is disproportionately low. For this reason, lanolin is considered a compound with relatively low allergenic potential. However, if we analyze the published reports and clinical cases devoted to lanolin allergy (Table 4), it is easy to see that the percentage of people allergic to this substance in certain groups of patients (such as those with atopic dermatitis or leg ulcers, small children, or the elderly) is higher than in the general population.

The term “lanolin paradox”, used to describe the unusual features of hypersensitivity to lanolin, was proposed in 1996 by R. Wolf [112]. It refers to the term “paraben paradox”, illustrating the surprising face of paraben allergy, which was proposed over 20 years earlier by A.A. Fisher [113].

R. Wolf [112] included four atypical features of lanolin allergy in the “lanolin paradoxes” (Table 9).

According to Wolf [112], the first and second paradoxes are caused by the fact that lanolin is probably a weak sensitizing agent. Therefore, healthy skin with a normal tissue structure is a sufficient barrier for this hapten. In the case of mechanically or inflammatorily damaged skin, lanolin penetrates deeply enough to trigger an immune system response, manifesting itself in a contact hypersensitivity reaction. Several studies have also noted that the use of skin-irritating substances (such as sodium lauryl sulfate or salicylic acid), together with lanolin and/or its derivatives, in patch tests increases the percentage of positive results [32,114,115]. It is also likely that the greater susceptibility of atopic skin to irritation, resulting from damage to the epidermal barrier and inflammation, may affect the results of patch tests with various substances. This causes slight differences in patch-test reaction patterns compared to non-atopic skin [116].

The third lanolin paradox is a direct result of the second lanolin paradox and addresses the fact that patch testing is performed on lesion-free skin. Moreover, according to Wolf [112], a lack of or low reactivity to lanolin in patch tests in patients with lanolin hypersensitivity may be the result of a low concentration of the sensitizing fraction of wool fat, which is most likely lanolin alcohols [6], found in the pure lanolin used in standard skin tests.

Wolf’s fourth lanolin paradox [112] results from the third paradox and was also observed by numerous researchers dealing with hypersensitivity to lanolin. Analysis of the data in Table 4 provides many such observations. A similar diagnostic problem was previously observed by Fisher [113] concerning paraben allergy, and he included these findings in the “paraben paradoxes”. He also suggested that using only a mixture of parabens in the diagnosis of hypersensitivity to these substances does not provide reliable results [113].

It is worth noting that Wolf’s “lanolin paradoxes” theory [112] also has opponents. An interesting example is the position reported by Kligman [105], according to which lanolin allergy is a myth that was created, mainly for commercial purposes, to increase demand for products marked as “lanolin-free”. According to this author, lanolin is, at best, a weak contact allergen and only affects a specific population. Kligman [105] believed that the risk of allergy to products containing lanolin is the result of erroneous scientific knowledge and a misunderstanding of the limitations of patch tests. Moreover, according to Kligman [105], no one has managed to sensitize animals or humans to lanolin or wool wax alcohols.

This statement does not seem to be entirely true, as Bourrinet and Berkovic [100,101] proved in their experiments, which induced hypersensitivity to lanolin and its derivatives in guinea pigs. It is also very interesting that earlier, Kligman [104] had been a strong supporter of the idea of lanolin allergy and pointed out the great importance of this problem.

Lanolin hypersensitivity has also been called a “wolf in sheep’s clothing” [4]. Johnson et al. [4] wanted to point out that despite the many advantages and benefits of using lanolin, we should not forget that wool fat can also be harmful. This is particularly important in those cases where its properties could be most useful (e.g., inflammation and/or skin damage). These authors also drew attention to the interesting fact that allergic contact dermatitis to wool fat is not related to an allergy to wool. Moreover, it is believed that wool allergy probably does not exist [117]. Wool causes non-immunological (non-allergic) irritating contact dermatitis, especially in atopic people [4].

## 7. Patch Tests for Lanolin Hypersensitivity—A Diagnostic Problem

The unusual nature of hypersensitivity reactions to lanolin (“lanolin paradoxes”) seems to result in difficulties in the diagnosis and diagnosis of contact allergy to wool fat and derivatives.

Diagnostic patch tests are performed on skin that is free from lesions. According to Wolf’s second “lanolin paradox” [112], in patients whose hypersensitivity to lanolin usually occurs at the site of application of medicinal and/or cosmetic products with lanolin, the results of patch tests on skin that is free from lesions may be falsely negative. These patients may tolerate the use of lanolin on healthy skin and develop hypersensitivity on inflamed skin [34,118]. In such a situation, it is possible that hypersensitivity to lanolin will not be confirmed in the diagnostic process.

The fourth “lanolin paradox”, according to Wolf [112], draws attention to the fact that the use of a single substance in epidermal patch tests for the diagnosis of contact hypersensitivity to lanolin may not be effective. Moreover, such a strategy may lead to both false negative and false positive results, and this is largely dependent on the condition of the patient’s skin at the time of testing. An analysis of the literature data presented in Table 4 of this review also allows us to notice that the use of additional patch tests using Amerchol L-101 significantly improves the quality of diagnostics for lanolin allergy. Matthieu and Dockx [61] analyzed routine patch test results in 393 patients diagnosed with contact dermatitis to investigate this issue (Table 10). Substances used in the patch tests were a standard mixture of lanolin alcohols (30%), Amerchol L-101 (100%), and (in 223 patients) Amerchol L-101 (50%). Based on the collected results, the authors noted that the observation that many lanolin allergy diagnoses could be missed in the case of contact allergy when using only a standard test with a mixture of lanolin alcohols (30%) seems to be accurate [61].

Although the validity of such a diagnostic strategy seems to be confirmed by the results of studies from other research teams [27,49,82], there may also be opinions questioning the advisability of testing with many lanolin derivatives. For example, according to Uldahl et al. [118], such a strategy may result in the overdiagnosis of lanolin allergy.

Among the diagnostic difficulties associated with diagnosing hypersensitivity to lanolin, many authors also point out that the reproducibility and repeatability of patch tests for this contact allergy is low. This problem is clearly illustrated by the research of the Bourke team [119] and the Ale team [120]. When Bourke et al. [119] analyzed the available literature data, they noticed that various inconsistencies in the results of this type of test were recorded in as many as 44% of cases. To verify these data, they conducted their own study, in which 383 patients underwent simultaneous, dual-patch tests on opposite sides of the upper back with 10 allergens from the European standard test series. Completely discordant patch test results, i.e., a negative test on one side and a positive test on the opposite side of the back, were recorded in 30 patients (i.e., 8%), including 28 for a single allergen and 2 for more than one allergen. For lanolin, non-compliance concerned 0.8% of the results. A similar study was conducted by Ale et al. [120], who performed simultaneous, dual-patch testing on opposite sides of the upper backs of 500 patients. They used 2 panels, each containing 12 standard allergens. A total of 435 positive patch test reactions were observed in 289 patients (i.e., 58.8%), on either one or both sides of the back. There were 22 (i.e., 5%) pairs of results that were discordant, i.e., they were interpreted as a positive result on one side and a negative or questionable result on the other side of the back. For lanolin alcohol, such an incompatibility was found in 1% of the results [120].

## 8. Summary and Conclusions

Hypersensitivity to lanolin and/or its derivatives, although not the only unusual phenomenon in dermatology [121,122], has been the subject of observations and studies for almost 100 years. Nevertheless, it still raises many questions and controversies. The significance and importance of this problem seems to be confirmed by the fact that the American Contact Dermatitis Society (ACDS) designated lanolin as the “contact allergen of 2023” [4]. The unusual nature of this allergy has been reflected in various attempts to understand and systematize this phenomenon. Hypersensitivity to lanolin has been called the “lanolin paradox”. There is also the theory of the “lanolin myth”, created for commercial purposes. Lanolin itself has been called “a wolf in sheep’s clothing”. These paradoxical properties of lanolin mean that it is not recommended for use on damaged skin, while, at the same time, it is considered useful for preventive skin care, helping to keep it in good condition.

The fact that lanolin is a substance that both causes and does not cause hypersensitivity in the same patient, depending on the skin’s condition, is a problem that researchers have been studying for decades. Despite many studies and analyses, this problem remains unsolved. Analysis of the available literature data allows us to assume that there is a lack of independent, well-developed programs in lanolin hypersensitivity studies. There is a lack of studies with well-selected, uniform groups of patients, conducted in standardized conditions. There is also a lack of studies concerning various, well-characterized lanolin preparations. Most of the available studies on the problem are studies based on retrospective analyses. In this type of study, neither the patient groups nor the preparations used are unified. This does not allow for the formulation of clear conclusions. The causative factor cannot be clearly determined. It is also impossible to use the results of these analyses to improve diagnostics and therapeutic procedures. Considering the continuing interest in the problem of lanolin hypersensitivity, the unusual nature of this reaction, and the diagnostic and therapeutic problems resulting from it, there seems to be a need to thoroughly investigate and understand all the mechanisms of this reaction, based on well-designed, extensive, standardized research projects.

## 9. Weaknesses, Limitations, and Possible Perspectives

This review is paradoxically limited by the seemingly large number of studies available for analysis in the field of lanolin hypersensitivity. Available publications are primarily retrospective analyses of test results performed during routine diagnostics or reports from clinical cases. In the context of an attempt to determine the sensitizing potential of lanolin and/or its derivatives, and the pathogenic mechanisms of hypersensitivity to these substances, the value and usefulness of this type of data are significantly limited. These data were collected from a mixed population, during a routine diagnostic process, by different doctors and in different medical facilities. The manner of conducting the clinical interview, the physical examination, the applied diagnostic strategies, test materials, and the manner of documenting medical data may differ. The collected clinical data are often incomplete in such cases (there is no standardized method of data collection), which may hinder coherent analysis. There is also a lack of independent studies involving healthy individuals. There is a lack of studies on cell models and possibly animal models. It seems that carefully planned, standardized, and reproducible studies, conducted under laboratory conditions, will be absolutely necessary to finally explain and understand the paradox of lanolin hypersensitivity.

## Figures and Tables

**Table 1:** Search terms used and preliminary results.

Search Term	Number of Results	Period Available for Analysis [Year of Publication From–To]
**PubMed**		
hypersensitivity to lanolin	150	1947–2024
allergy to lanolin	178	1947–2024
hypersensitivity to lanolin derivatives	22	1980–2024
allergy to lanolin derivatives	26	1976–2024
amerchol	45	1979–2024
amerchol hypersensitivity	28	1997–2024
amerchol allergy	33	1979–2024
**Google Scholar**		
hypersensitivity to lanolin (pdf)	About 4070 results	not limited
allergy to lanolin (pdf)	About 8900 results	not limited
hypersensitivity to lanolin derivatives (pdf)	About 1590 results	not limited
allergy to lanolin derivatives (pdf)	About 2820 results	not limited
amerchol (pdf)	About 1420 results	not limited
amerchol hypersensitivity (pdf)	About 631 results	not limited
amerchol allergy (pdf)	About 909 results	not limited
responsible allergens in lanolin (pdf)	About 3840 results	not limited
allergens in lanolin (pdf)	About 9580 results	not limited

**Table 2:** Percentage composition of the various forms of lanolin [13].

Component	Raw Lanolin	Lanolin Oil (Liquid)	Lanolin Wax (Solid)
Esters of sterols and triterpene alcohols [%]	35.4	44.0	28.9
Esters of aliphatic alcohols [%]	23.7	16.0	13.9
Monohydroxyesters of sterols and triterpenes and aliphatic alcohols [%]	20.0	15.0	16.4
Di- and polyhydroxyesters and free diols [%]	7.9	7.7	9.3
Free aliphatic alcohols [%]	5.6	10.4	20.2
Free sterols [%]	4.1	4.4	5.3
Free hydrocarbons [%]	0.6	0.3	0.4
Free fatty acids [%]	0.5	0.7	1.0
Other substances [%]	2.2	1.5	4.6

**Table 3:** Main features and applications of lanolin oil and wax [14].

Lanolin Oil	Lanolin Wax
It is characterized by clarity, is less sticky, and has better spreading properties on the skin and less adhesion than raw lanolin. It serves as a plasticizer and/or dispersant in cosmetics/medicines. It is typically used for lotions, face creams, baby oils, hair oils, suntan oils, lipsticks, and lip glosses.	It is a better water/oil emulsifier than raw lanolin. In cosmetics/medicines, it acts as a stabilizing and binding substance that increases the cohesion and homogeneity of wax mixtures, creams, and lipsticks. It is usually used in creams, lipsticks, and lip glosses. It is completely odor- and taste-free.

**Table 4:** Allergy/hypersensitivity to lanolin: a review of the reports on lanolin allergy published between 1950 and 2024, based on a search of the PubMed database with the queries “lanolin allergy” [35] and “lanolin hypersensitivity” [36].

Author’s (Year of Publication) [No. Reference]		Brief Description and/or Main Features of the Study
Sulzberger M.B. and Lazar M.P. (1950) [6]	Study group:	Four people with eczema contact hypersensitivity
	Methods:	Epidermal patch tests with lanolin (from various sources) and its derivatives (alcohols and lanolin acids)
	Results/conclusions:	All patients reacted to mixed lanolin alcohols, one patient also reacted to lanolin
Sulzberger M.B. et al. (1953) [7]	Study group:	1048 people with various skin lesions and 120 healthy people
	Methods:	screening: epidermal patch tests with anhydrous lanolin people with a positive screening result: epidermal patch tests with 16 substances containing lanolin and/or its derivatives, including bases for medicinal ointments, creams, and cosmetics
	Results/conclusions:	The frequency of positive results in the lanolin patch tests was 1.14% and they assumed that the aliphatic lanolin alcohol fraction was mainly responsible for allergic reactions
Warshaw T.G. (1953) [37]	Study group:	1430 patients with various types of skin lesions
	Methods:	Epidermal patch tests with anhydrous lanolin
	Results/conclusions:	The frequency of positive results in the lanolin patch tests was 1.05%
Baer R.L. et al. (1955) [38]	Study group:	637 patients with contact dermatitis
	Methods:	Epidermal patch tests with lanolin
	Results/conclusions:	The frequency of positive results in lanolin patch tests was 4.4%
Hjorth N. (1959) [39]	Study group:	550 people with a documented allergy to cosmetics
	Methods:	Epidermal patch tests with lanolin
	Results/conclusions:	The frequency of positive results in lanolin patch tests was 3.8%
Bandmann H.J. and Reichenberger M. (1957) [40]	Study group:	4000 people with a contact rash
	Methods:	Epidermal patch tests with Eucerin
	Results/conclusions:	The frequency of positive results in Eucerin patch tests was 0.25%
Hjorth N. and Trolle-Lassen C. (1963) [41]	Study group:	People with various types of skin lesions
	Methods:	Epidermal patch tests with various lanolin derivatives
	Results/conclusions:	The frequency of positive patch tests was: Eucerin: 2.07% (39 positive results out of 1878 people tested) A mixture of lanolin and its derivatives: 2.28% (38 positive results out of 1664 people tested) Lanolin alcohols: 1.6% (27 positive results out of 1664 people tested)
Wereide K. (1965) [42]	Study group:	512 people with various types of contact skin reactions
	Methods:	Epidermal patch tests with Eucerin
	Results/conclusions:	The frequency of positive results in the Eucerin patch tests was 10.7%
Reichenberger M. (1965) [43]	Study group:	150 people with leg ulcers
	Methods:	Epidermal patch tests with Eucerin
	Results/conclusions:	The frequency of positive results in patch tests with Eucerin was 18.6%
Thune P. (1969) [32]	Study group:	230 people with leg ulcers
	Methods:	Epidermal patch tests with anhydrous lanolin, anhydrous Eucerin
	Results/conclusions:	The frequency of positive patch tests was: Anhydrous lanolin: 2.17% Anhydrous Eucerin: 2.61%
Vollum D.I. (1969) [44]	Study group:	Two clinical cases of skin hypersensitivity, in the form of erythema, vesicles, and edema, to steroid ointment containing 10% hydrogenated lanolin (case report)
	Methods:	Epidermal patch tests with lanolin
	Results/conclusions:	In both cases, patch tests with lanolin were positive.
Epstein E. (1972) [45]	Study group:	298 patients with various types of skin lesions
	Methods:	Epidermal patch tests with lanolin alcohols at a concentration of 30%
	Results/conclusions:	The frequency of positive patch tests with lanolin alcohols was 2.4%
Agelini G. et al. (1975) [46]	Study group:	306 patients treated conservatively for stasis dermatitis with or without ulceration
	Methods:	Epidermal patch tests with 63 different substances (active and auxiliary) that are present in various topical preparations (including lanolin)
	Results/conclusions:	Positive reactions to one or more of the tested compounds were recorded in 177 patients. The main excipients that gave positive results were parabens, lanolin, and benzoyl peroxide
Sugai T. and Higashi J. (1975) [47]	Study group:	502 patients with hypersensitivity to steroid ointment
	Methods:	Epidermal patch tests with lanolin and hydrated lanolin
	Results/conclusions:	The frequency of positive patch tests was: Lanolin: 1.99% Hydrogenated lanolin: 5.02% Lanolin and hydrogenated lanolin (simultaneously): 0.5%
Hannuksela M. et al. (1976) [48]	Study group:	Eczema patients
	Methods:	Hypersensitivity to common ingredients of cosmetics and drugs, such as fragrances, antibacterial agents, emulsifiers, surfactants, propylene glycol, lanolin, and wool alcohols, tested over a period of three years
	Results/conclusions:	The frequency of allergy to lanolin and lanolin alcohols was 1.2%
Mortensen T. (1979) [49]	Study group:	Series 1 study: 1230 patients with eczema Series 2 study: 899 patients with eczema
	Methods:	Series 1 of tests: epidermal patch tests with lanolin alcohols Series 2 tests: epidermal patch tests with lanolin alcohols, hydrogenated lanolin (30%) in soft yellow paraffin, Amerchol L-101, and a mixture of various lanolin derivatives
	Results/conclusions:	Series 1 of the study: the frequency of positive reactions to lanolin alcohols was 2.7% Series 2 of the study: the frequency of positive results for lanolin and/or its derivatives was 6.6%
von Liebe V. et al. (1979) [50]	Study group:	A 42-year-old woman with contact allergy to lanolin on the skin of her hands, which occurred as a result of occupational exposure—hairdresser (case report)
	Methods:	Open epidermal patch tests with cosmetics to which she was exposed at work and their ingredients with known sensitizing potential (including lanolin derivatives)
	Results/conclusions:	30 min after applying the tested substances, the patient experienced positive urticarial reactions to the permanent wave solution, fixative solution, p-aminodiphenylamine, lanolin alcohol, and clioquinol
Jenni C. and Zala L. (1980) [51]	Study group:	60 patients with dermatitis of the lower legs
	Methods:	Standard epidermal patch tests
	Results/conclusions:	More than 50% of patients with dermatitis of the lower legs had a contact allergy. The most common allergens in this group of patients were: balsam of Peru, lanolin and turpentine, benzocaine, p-phenylenediamine, neomycin, oxyquinoline, and parabens
Förg T. et al. (1982) [52]	Study group:	3300 women with contact dermatitis
	Methods:	Retrospective analysis (period: 1970–1974) of patch tests results
	Results/conclusions:	Lanolin alcohols were indicated as one of the main allergens found in various articles in the field of so-called household chemicals
Frenzel U. and Gutekunst A. (1985) [53]	Study group:	133 patients with contact dermatosis of leg ulcers
	Methods:	Analysis of the allergenic effect of six substances: lanolin, neomycin, balsam of Peru, parabens, phenylenediamine and benzocaine; patch tests
	Results/conclusions:	Lanolin is one of the most common contact allergens in this group of patients
Edman B. (1985) [54]	Study group:	1016 patients with various types of eczema
	Methods:	Retrospective study covering a period of 2 years; computer analysis of correlations between the location of eczema and individual contact allergens (including lanolin and/or its derivatives)
	Results/conclusions:	The study confirms, among others, a correlation between lanolin allergy and lower leg dermatitis
Wilson C.L. et al. (1991) [55]	Study group:	81 patients with venous leg ulcers
	Methods:	Retrospective study covering a period of 11 months; analysis of the results of standard epidermal patch tests
	Results/conclusions:	Positive patch tests were observed in 54 patients (67%), including a persistently high incidence of allergy to lanolin and topical antibiotics. Multiple allergies were diagnosed in 48 patients (58%)
Lever R. and Forsyth A. (1992) [56]	Study group:	73 adult patients with atopic dermatitis
	Methods:	Epidermal patch tests
	Results/conclusions:	A positive reaction (to at least one allergen) occurred in 42% of patients (predominantly women); the most frequently identified allergens were fragrances, nickel (9.6%), rubber (6.8%), lanolin (5.5%), and formaldehyde (4.1%)
Pasche-Koo F. et al. (1994) [57]	Study group:	47 patients with chronic or recurrent (>1 year) inflammatory skin disease (leg ulcers, contact dermatitis, atopic dermatitis, or psoriasis)
	Methods:	Epidermal patch tests with: with the following emulsifiers: Atlas G 1441 (polyoxyethylenesorbitan lanolin derivative), Atlas G 2162 (polyoxyethyleneoxypropylene stearate), Arlacel 83 (sorbitansesquioleate), Tween 40 (polyoxyethylenesorbitanmonopalmitate), Lanette N, Lanette O (cetylstearyl alcohol), Span 60 (sorbitan monostearate), Span 80 (sorbitan monooleate), triethanolamine, Tween 80 (polyoxyethylenesorbitan monooleate) Epidermal tests using own medicines/cosmetics for topical use or ready-made dressings (also contained lanolin)
	Results/conclusions:	12 patients (including 10 with leg ulcers) had at least 1 positive reaction (also with lanolin) after 3 or 4 days 6 patients with leg ulcers had positive reactions with their own medicinal and cosmetic products and/or dressings
Dotterud L.K. and Falk L.S. (1995) [58]	Study group:	424 children (aged 7–12) from northern Norway
	Methods:	Standard epidermal patch tests
	Results/conclusions:	The most common allergen was nickel (14.9%), followed by cobalt (5.7%), catone CG (5.2%), lanolin (1.7%), and neomycin (1.4%)
Van Ginkel C.J. et al. (1995) [59]	Study group:	34 patients suffering from chronic ear discharge
	Methods:	Standard epidermal patch tests
	Results/conclusions:	Lanolin (found in the bases of ear ointments and creams) was one of the allergens reacting in the patch tests
Schauder S. and Ippen H. (1997) [60]	Study group:	402 patients reporting dermatoses and photodermatoses after using preparations containing sunscreens
	Methods:	Retrospective study (period: 1981–1996); the results of epidermal patch tests were analyzed
	Results/conclusions:	Lanolin alcohols accounted for 3.3% of positive tested results
Matthieu L. and Dockx P. (1997) [61]	Study group:	393 patients consulted the dermatologist for dermatitis (country: Belgium)
	Methods:	Retrospective study (period: April 1991 to February 1992); analysis of the results of patch tests (393 patients) with a standard series (containing wax-wool alcohols: 30% pet.; Chemotechnique Diagnostics AB) and Amerchol L-101 100% (containing 10% wax-wool alcohols; Chemotechnique Diagnostics AB) and (223 patients) Amerchol L-101 50% pet. (contains 5–10% wax-wax alcohols; Trolab—cat. no. E0020)
	Results/conclusions:	3.1% of patients showed positive test results for wax alcohols (30%) and Amerchol L-101 (100%); 0.3% only for wax alcohols (30%); 13.5% only for Amerchol L-101 (100%); 12.1% on Amerchol L-101 100% and/or 50%; 3.6% only for Amerchol L-101 50%. Diagnosis of hypersensitivity to lanolin using one test substance was ineffective
Le Coz C.J. et al. (1998) [62]	Study group:	50 patients with leg ulcers of variable duration (15 days to 32 years, median 2 years)
	Methods:	Epidermal patch tests (European standard series; including Amerchol L101 and lanolin alcohols)
	Results/conclusions:	76% of patients showed positive results for at least one of the tested substances, including 18% who reacted to Amerchol L101 and 14% to lanolin alcohols
Reichert-Pénétrat S. et al. (1999) [63]	Study group:	359 patients hospitalized for venous and/or arterial leg ulcers, with or without the clinical occurrence of peri-ulcerative contact dermatitis
	Methods:	Standard ICDRG (International Contact Dermatitis Research Group) patch tests and a specific series of 40 tests
	Results/conclusions:	Positive patch tests were observed in 82.5% of patients. The most common causes of positive results were balsam of Peru, lanolin, and neomycin
Giordano-Labadie F. et al. (1999) [64]	Study group:	114 children under 16 years of age with atopic dermatitis
	Methods:	Epidermal patch tests based on a series of European standards, with thixocortol, pivalate, budesonide, and the emollient used
	Results/conclusions:	Contact allergy was found in 43% of the examined children; the most common allergens were metals (19.3%), fragrances (4.4%), balsam of Peru (2.6%), lanolin (4.4%), neomycin (2.6%), and emollients (2.6%).
Wakelin S.H. et al. (2001) [65]	Study group:	24,449 patients with various types of skin lesions
	Methods:	retrospective study (period: 1982–1996); the results of epidermal patch tests were analyzed (standard series, containing wool alcohols (30%))
	Results/conclusions:	The average annual lanolin sensitivity rate was 1.7%; in the group of people allergic to lanolin alcohols, there was a higher percentage of women; the highest incidence of allergy to wool alcohols was found in patients with dermatitis of the lower legs (6.0%), followed by patients with dermatitis of the anogenital area (3.23%); the percentage of tests with AmercholL101 was lower than with wool alcohol, with some patients who had positive results for Amerchol also having negative results for lanolin alcohols; the average annual allergy rate to Eucerin was 0.65% and to hydrogenated lanolin in petroleum jelly in a 1:1 ratio was 1%
Machet L. et al. (2002) [66]	Study group:	106 patients with leg ulcers
	Methods:	Retrospective study (two-year period); analysis of medical documentation, including the results of patch tests with the European standard series and additional series
	Results/conclusions:	75% of 106 patients had at least one positive reaction and 57% had two or more positive reactions; the frequency of allergy to lanolin was 21%; lanolin was the second most common allergen in this group of patients, after balsam of Peru (40%)
DeleoV.A. et al. (2002) [67]	Study group:	9624 patients diagnosed with allergic contact dermatitis or irritant contact dermatitis (black (*n* = 1014) and white (*n* = 8610)); study of allergic contact dermatitis or irritant contact dermatitis, examined by members of the North American Contact Dermatitis Group between 1992 and 1998
	Methods:	Retrospective study (period: from July 1, 1992 to June 30, 1998); analysis of epidermal patch tests results (41 allergens)
	Results/conclusions:	Black patients showed a higher rate of allergy to paraphenylenediamine, cobalt chloride, thioureas, and p-tert-butylphenol-formaldehyde resin White patients showed higher rates of allergy to formaldehyde, glutaraldehyde and some formaldehyde-releasing preservatives, as well as to lanolin, epoxy resin, thiourea, and balsam of Peru
Kiec-Swierczynska D. et al. (2003) [68]	Study group:	132 farmers suspected of contact allergy as an occupational disease (country: Poland)
	Methods:	Epidermal patch tests with a standard set of allergens (Chemotechnique Diagnostic AB, Malmö, Sweden) containing lanolin and a set of allergens for farmers, developed by the authors (contains 39 different agricultural chemical substances)
	Results/conclusions:	The frequency of allergy to lanolin (lanolin alcohols) was 5.3%
Machovcova A. et al. (2005) [69]	Study group:	12,058 patients with suspected contact dermatitis (country: Czech Republic)
	Methods:	Retrospective study (period: January 1997 to December 2001); analysis of the results of epidermal patch tests (23 allergen-tested panel Trolab (Hermal, Reinbeck, Germany)
	Results/conclusions:	7661 (63.5%) patients experienced 1 or more positive reactions; the frequency of an allergy to lanolin (lanolin alcohols) was 3.0%
Goon A.T. and Goh L. (2006) [70]	Study group:	2340 patients with contact dermatitis (age, less than 21 years; country, Singapore)
	Methods:	Retrospective epidemiological study of allergic contact dermatitis in children and adolescents (period: from 1 January 1986 to 31 December 2003); the results of the epidermal patch tests were analyzed
	Results/conclusions:	Most positive results in the patch tests occurred for: nickel (40%), thiomersal (15%), rosin (9%), lanolin (8%), cobalt (8%), fragrance mixture (5%), and neomycin (4%)
Oppel T. and Schnuch A. (2006) [71]	Study group:	9948 patients with suspected contact allergy (country: Germany)
	Methods:	Retrospective study (period: January to December 2004); analysis of the results of epidermal patch tests for 10 allergens considered to be the most common allergens in Germany
	Results/conclusions:	Nickel sulfate (17.2%), fragrance mixture (7.2%), balsam of Peru (6.7%), cobalt chloride (6.5%), potassium dichromate (5.3%), rosin (4.6%), lanolin alcohol (4.3%), p-phenylenediamine (4.2%), ammonium mercury (3.5%), and methyldibromoglutaronitrile/phenoxyethanol (3.4%)
Tomljanović-Veselski M. et al. (2007) [72]	Study group:	60 patients (including 30 patients with leg ulcers and 30 without allergic contact dermatitis).
	Methods:	Both groups were tested for allergens of the standard series and allergens of the target series, as well as topical agents most often used by patients
	Results/conclusions:	Leg ulcer group: 57 positive reactions with allergens of both series were observed; the most common allergic reactions were to balsam of Peru, fragrance mixture and neomycin sulfate Group without allergic contact dermatitis: 45 positive reactions with allergens of both series were observed; the most common allergic reactions were to corticosteroid ointments, lanolin, and bepanthene The frequency of allergy to lanolin was the same in both groups and amounted to 6.7%
Beattie P.E. et al. (2007) [73]	Study group:	79 children with suspected contact dermatitis(country: Great Britain)
	Methods:	Epidermal patch tests (T.R.U.E. TEST^®^ (SmartPractice Denmark, Hillerød, Dania) and thixocortol 17-pivalate, budesonide, and 3 emollients)
	Results/conclusions:	51% had 1 or more positive allergic patch tests reactions; the frequency of allergy to lanolin (lanolin alcohols) was 4.5%
Smart V. et al. (2008) [74]	Study group:	100 patients with chronic venous disease and other causes of leg ulcers
	Methods:	Epidermal patch tests with 38 common contact allergens (including those considered important in patients with leg ulcers)
	Results/conclusions:	Overall, 46% of patients had at least 1 positive patch test response. Multiple reactions in the same patient were common; the most common allergic reactions were to fragrances, lanolin, antibacterial agents, and rubber allergens
Hogeling M. and Pratt M. (2008) [75]	Study group:	100 patients (age 4–18 years) with suspected contact dermatitis (country/city: Canada/Ottawa)
	Methods:	Retrospective study (period: 1996–2006); analysis of epidermal patch tests results
	Results/conclusions:	70% of children had at least one positive reaction in the patch tests, of which 4% were positive results for lanolin
Warshaw E.M. et al. (2009) [76]	Study group:	26,479 patients (country: United States of America; USA)
	Methods:	Retrospective study; analysis of the results of epidermal patch tests (research conducted by the North American Contact Dermatitis Group (NACDG) in 1994–2006)
	Results/conclusions:	2.5% of patients had positive reactions to lanolin alcohol at 30% in Vaseline; the incidence of lanolin allergy decreased from 3.7% in 1996–1998 to 1.8% in 2005–2006; positive reactions were observed more often in women than in men; lanolin allergy was more common in patients with atopic dermatitis; cosmetics were usually the source of lanolin; 2.5% were related to occupational exposure; allergy to lanolin increased the likelihood of a positive reaction to another of the tested contact allergens
Schnuch A. et al. (2009) [77]	Study group:	18,572 patients with facial dermatitis (study country: Germany)
	Methods:	Retrospective study (period: 1995–2007); analysis of epidermal patch tests results
	Results/conclusions:	An allergy to lanolin alcohols was found in 3.0% of women and 2.2% of men; the main sources of exposure are cosmetics (mainly women) and occupational exposure (mainly men)
Nguyen J.C. et al. (2010) [78]	Study group:	90-year-old woman (case report); skin reaction to Aquaphor (Beiersdorf; Wilton, Connecticut), a water-in-oil emollient. Its main ingredients are 43% Vaseline, mineral oil, ceresin wax (mineral wax), and lanolin alcohol (wool)
	Methods:	Patch testing on the inside of the right arm using the Finn chamber technique, with Aquaphor and the individual ingredients contained in this product (i.e., white petrolatum, mineral oil, ceresin wax, and lanolin alcohol)
	Results/conclusions:	The patient showed a quick, positive reaction to Aquaphor and lanolin alcohol
Minamoto K. (2010) [79]	Study group:	1556 patients with various types of contact lesions on the skin (country: Japan)
	Methods:	Retrospective study (period: 1994–2003); analysis of epidermal patch tests results
	Results/conclusions:	The frequency of allergy to lanolin (lanolin alcohols) was 2.7%; the main sources of lanolin were emollients
Beliauskienė A. et al. (2011) [80]	Study group:	35 patients with chronic leg ulcers and surrounding dermatitis and 59 patients with contact dermatitis of the legs and feet
	Methods:	Epidermal patch tests with allergens from the European base series
	Results/conclusions:	80% of patients with chronic leg ulcers and 41% of patients with dermatitis of the lower leg or foot had at least one positive test result; allergy to lanolin alcohol, benzocaine and p-phenylenediamine was more common in patients with chronic leg ulcers; the frequency of allergy to lanolin alcohols in this group of patients was 17%
Fellinger C. et al. (2013) [81]	Study group:	19-year-old woman with a severe skin reaction (blisters, disseminated eruptions, and swelling in the hands, cheeks, and feet) to propolis cream (case report)
	Methods:	Epidermal patch tests with a base of an own-series fragrance mixture of series I and II (Almirall Hermal, Reinbek, Germany) and Curatest^®^ (Lohmann and Rauscher, Rengsdorf, Germany), various ointment bases (solid paraffin, liquid paraffin, cetearyl alcohol 20% pet., and Vaseline) and products indicated by the patient (horse hoof ointments, bay leaf oil (ointment ingredient), propolis cream (composition: lanolin alcohol, cetearyl alcohol, polyethoxylated castor oil, solid paraffin, liquid paraffin, petroleum jelly, beeswax, olive oil, and propolis), and the patient’s perfume)
	Results/conclusions:	During the diagnostic process, it was determined that the factor that caused the changes was lanolin alcohol, which was a component of the propolis cream base
Miest R.Y. et al. (2013) [82]	Study group:	286 patients with suspected allergic contact dermatitis due to hypersensitivity to lanolin
	Methods:	Epidermal patch tests with: lanolin alcohol (30% in Vaseline), Amerchol L101 (50% in Vaseline), 10 lanolin derivatives
	Results/conclusions:	The frequency of positive reactions to lanolin in this group of patients was determined to be 6.29%
Fraser K. and Pratt M. (2015) [83]	Study group:	27-year-old woman with recurrent lip dermatitis (case report)
	Methods:	Epidermal patch tests with selected substances
	Results/conclusions:	Positive reactions to thixocortol-21-pivalate (3+), lanolin (3+), neomycin (3+), nickel (1+), hydroxyethyl methacrylate (3+), bacitracin (3+), and abitol (3+), and also for lip balms containing lanolin and topical products containing hydrocortisone and bacitracin
Warshaw E.M. et al. (2015) [84]	Study group:	4283 patients with suspected contact dermatitis (country: United States of America)
	Methods:	Retrospective study; analysis of the results of epidermal patch tests for 70 contact allergens (standardized series) from 12 centers in the USA (this study documents the results of tests conducted by the North American Contact Dermatitis Group (NACDG) from 1 January 2011 to 31 December 2012)
	Results/conclusions:	63.8% had at least one positive reaction; the frequency of allergy to lanolin was estimated at 4.6%; in 9.6% of cases, the cause of contact skin allergy was related to work
BelloniFortina A. et al. (2015) [85]	Study group:	Study group: 6008 patients aged 1–16 with suspected allergic contact dermatitis
	Methods:	Retrospective study; analysis of the results of epidermal patch tests collected by the European Surveillance System on Contact Allergies (ESCA) from 11 European countries (period: 2002–2010)
	Results/conclusions:	The overall incidence of at least one positive reaction to hapten was 36.9%; the 10 most frequent allergens were nickel sulfate (16.7%), cobalt chloride (7.5%), potassium dichromate (5.2%), neomycin sulfate (3.2%), Myroxylonpereirae resin (2.6%), para-phenylenediamine (PPD, 2.5%), chloromethylisothiazolinone/methylisothiazolinone 3:1 (MCI/MI, 2.4%), fragrance mix (2.3%), lanolin alcohols (1.8%), and colophony (1.4%).
Mahler V. (2015) [86]	Study group:	14,841 patients over 65 years of age with skin lesions (country: Germany)
	Methods:	Retrospective study (period: 2009–2013), analysis of the results of epidermal patch tests; data source: Information Association of Dermatology Clinics (IVDK)
	Results/conclusions:	The 10 most frequently recognized contact allergens in people over 65 years of age were fragrance mixtures, balsam of Peru, nickel (II) sulfate, fragrance mixture II, rosin, propolis, chloromethylisothiazolinone/methylisothiazolinone (3:1), wool wax alcohols, Amerchol L 101, tert.-butylhydroquinone
Higgins C.L. and Nixon R.L. (2016) [87]	Study group:	52-year-old man suffering from atopy, with a 12-month history of eye pain and irritation, along with eyelid erythema and patchy facial dermatitis (case report)
	Methods:	Epidermal patch tests, performed according to the criteria of the International Contact Dermatitis Study Group, using the Australian baseline series, common ocular allergens, and the patient’s own samples (medicated ointments and cosmetics)
	Results/conclusions:	On the second and fourth days, there were positive reactions to lanolin derivatives: wool alcohols (30% pet.), and Amerchol L-101 (50% pet.) (Chemotechnique, Vellinge, Sweden); no other positive reactions occurred; lanolin was identified in the composition of Refresh Lacri-Lube Lubricant Eye Ointment (Allergan, Sydney) nocte, containing 0.2% *w*/*w* wool alcohols, which was used by the patient
Uter W. et al. (2016) [88]	Study group:	58,833 patients tested in 12 European countries (54 centers) due to suspected contact allergy
	Methods:	Retrospective study (period: 2009–2012); analysis of data (results of epidermal patch tests) collected by the European Surveillance System for Contact Allergies (ESSCA)
	Results/conclusions:	Positive reactions were most frequently observed to sodium metabisulfite (3.12%), followed by propolis (2.48%), Compositae mixture (1.73%), lanolin alcohols (1.65%) and kain mixture III (1.27%)
Erfurt-Berge C. et al. (2017) [89]	Study group:	Study group: 5264 patients with dermatitis of the lower legs, chronic venous insufficiency, or chronic leg ulcers
	Methods:	Retrospective analysis (period: 2003–2014) of epidermal patch tests results
	Results/conclusions:	The frequency of allergy to lanolin in this group of patients was estimated at 7.8% (in tests with lanolin alcohols) and 9.7% (in tests with Amerchol L-101)
Lubbes S. et al. (2017) [90]	Study group:	1012 patients of <18 years of age, diagnosed with suspected contact dermatitis (country: The Netherlands)
	Methods:	Retrospective study (period: 1996–2013); analysis of epidermal patch test results; patch tests were performed in van der Bend chambers (van der Bend, Brielle, The Netherlands), in combination with allergens from Almirall (Reinbek, Germany) or Chemotechnique (Vellinge, Sweden), or the TRUE Test^®^ assay (SmartPractice Denmark, Hillerød, Denmark)
	Results/conclusions:	46% of children had at least one positive reaction; positive reactions to lanolin alcohol (30%) occurred in 66% of children with atopic dermatitis and 29% in children without atopic dermatitis; for Amerchol L-101, it was 59% and 14%, respectively
Jacob S.E. et al. (2017) [91]	Study group:	1142 children diagnosed with atopic dermatitis and skin lesions with other causes
	Methods:	Retrospective study; (period: from 1 January 2015 to 31 December 2015); the results of epidermal patch tests were analyzed; all studies were performed in the United States
	Results/conclusions:	In children with atopic dermatitis, there was a statistically significantly increased frequency of reactions to cocamidopropyl betaine, wool alcohol, lanolin, thixocortol pivalate, and parthenolide, with a lower frequency of reactions to methylisothiazolinone, cobalt, and potassium dichromate; the frequency of allergy to lanolin was 26%
Uter W. et al. (2018) [26]	Study group:	130,510 patients with suspected contact allergy (country: Germany)
	Methods:	Retrospective study (period: between 2006 and 2016); analysis data of all patients patch-tested with Amerchol^®^ L101 50% pet., included in a special test series (63% of patients), or with lanolin alcohols 30% pet., tested in the baseline series of the German Contact Dermatitis Research Group (DKG; https://dkg.ivdk.org/) (88% of patients)
	Results/conclusions:	Reactions of ++ or +++ intensity accounted for less than one-quarter of positive reactions (except for patients with leg dermatitis, where it accounted for almost 25% of all positive reactions; the percentage of all positive results in this group of patients was the highest); the estimated percentage of positive reactions to the Amerchol^®^ L101 test was slightly higher than the percentage of positive reactions to lanolin alcohols (2.4% vs. 2.38%)—Amerchol^®^ L101 caused more positive patch-test reactions than lanolin alcohols in aimed testing
Fransen M. et al. (2018) [92]	Study group:	9577 patients with dermatitis
	Methods:	Retrospective study (from 1 January 2004 to 31 December 2015); the results of epidermal patch tests using 30% pet. lanolin alcohols were analyzed. and Amerchol™ L-101 50% pet.
	Results/conclusions:	An increase in the frequency of lanolin allergies was observed from 0.45% in 2004 to 1.81% in 2015; weak, significant associations were found between atopic dermatitis and allergy to lanolin and lanolin alcohols (no relationship was found with Amerchol™ L-101); 1.2% of patients had a positive reaction to lanolin alcohols or Amerchol™ L-101; 0.3% reacted to both substances
Pap E.B. et al. (2018) [93]	Study group:	100 patients aged 13–18 undergoing diagnoses of skin lesions; 47% were adolescents diagnosed with atopic dermatitis (country: Hungary)
	Methods:	Retrospective study (period: January 2007 to December 2016); analysis of epidermal patch-tests results (EEBS European series)
	Results/conclusions:	Positive patch tests for at least one allergen were found in 51% of teenagers; among the tested allergens in the entire group, the most common allergens were: nickel sulfate (17%), thimerosal (12%), para-phenylenediamine (PPD) (8%), cobalt (7%), fragrance mixture I (6%), and lanolin (5%); the most common contact allergens in people with atopic dermatitis were nickel (12.8%), lanolin (10.6%), thiomersal (8.5%), MCI/MI (6.4%), and fragrance mixture I (6.4%); lanolin allergy was more common in boys (7.7%) than in girls (4.1%)
Rastogi S. et al. (2018) [94]	Study group:	502 adults (aged ≥ 18 years) diagnosed with atopic dermatitis; AD was diagnosed in 40% of respondents (country: USA)
	Methods:	Retrospective study (period: 2014–2017); analysis of the results of epidermal patch tests with an extended series of allergens
	Results/conclusions:	Lanolin was one of the most common allergens, especially in the group with active atopic dermatitis
Knijp J. et al. (2019) [27]	Study group:	594 patients with suspected contact allergies (country: The Netherlands)
	Methods:	Retrospective study (period: 2016–2017); analysis of data from patch tests with lanolin alcohol 30% pet., Amerchol L101 50% pet., and a supplementary series containing other lanolin derivatives (lanolin alcohol and Amerchol L101 were tested in duplicate)
	Results/conclusions:	28.6% (95% confidence interval [CI]: 25.1–32.3%) had a positive patch test reaction to at least one lanolin derivative; reactions to lanolin alcohol (14.7%, 95% CI: 11.3–18.2%) and Amerchol L101 (15.0%, 95% CI: 11.5–18.5%) were common in the routinely tested series; reactions to other test preparations (lanolin cosmetics) were significantly less frequent; and the addition of Amerchol L101 to lanolin alcohol significantly increased the number of positive cases (odds ratio 1.79, *p* < 0.001)
Silverberg J.I. et al. (2022) [33]	Study group:	43,691 patients with suspected contact allergies (country: USA)
	Methods:	Retrospective study (period: 2001–2018); analysis of the results of epidermal patch tests for lanolin: lanolin alcohol 30% or Amerchol L-101 (50% in Vaseline) (study conducted by the North American Contact Dermatitis Group)
	Results/conclusions:	The frequency of allergy to lanolin was estimated at 4.63%; the most common anatomical locations of dermatitis were the hands (20.7%), diffuse/generalized distribution (19.6%), and face (17.0%); allergy to lanolin occurred more often in children (4.5%) than in adults (3.2%); the main sources of lanolin were personal care products and medicines; 2.24% of allergies were related to work
Németh D. and Pónyai G. (2022) [95]	Study group:	600 patients (patient age > 60 years) with suspected contact dermatitis (country: Hungary)
	Methods:	Epidermal patch tests with the European Environmental Baseline Series (EEBS) and Complementary Fragrance Series (CFS) allergens
	Results/conclusions:	The frequency of allergy to lanolin was: 4.7% (women), 5.5% (men)
Németh D. et al. (2022) [96]	Study group:	5790 patients (age > 18 years) with contact hypersensitivity, including 629 patients with atopic dermatitis (AD) (country: Hungary)
	Methods:	Retrospective study; (period: 2007–2021); analysis of the results of epidermal patch tests (standard European EEBS series); the focus was only on the group of patients diagnosed with atopic dermatitis
	Results/conclusions:	Lanolin was one of the five most common contact allergens in this group of patients (a positive result in tests with lanolin alcohol (30%) was observed in 20% of patients with atopic dermatitis)

**Table 5:** Groups of patients at high risk of developing hypersensitivity to lanolin.

Patients Particularly Susceptible to Hypersensitivity to Lanolin:
People using products containing lanolin and/or its derivatives (medicines/cosmetics) on damaged or inflamed skin (e.g., patients with atopic dermatitis—in the skin lesions, and patients with leg ulcers of various origins, including venous congestive disease—in the skin lesions)
People using products containing lanolin and/or its derivatives (medicines/cosmetics) in the area of inflammatory lesions (e.g., in the ear, conjunctival, or intranasal)
People using products containing lanolin and/or its derivatives (medicines/cosmetics) in dressings on mechanically damaged skin (e.g., after surgery)—occlusive dressings in damaged skin
People exposed to lanolin and/or its derivatives, in connection with their professional work
People exposed to lanolin and/or its derivatives, due to significant/frequent or long-term exposure to household chemicals
Children and people over 65 years of age
Female persons, regardless of age

**Table 6:** Clinical characteristics of the patients included in the Sulzberger and Lazar study [6].

Patient (P)	Sex	Relevant Clinical Data	Source of Lanolin (Cause of Symptoms)
P1	Female	Allergic eczematous contact-type dermatitis on the skin around the lips, a form of cheilitis exfoliativa	Lipstick
P2	Female	Allergic eczematous contact dermatitis of local localization, limited to the area where medicated ointments and/or creams were applied	Various medicated ointments and/or creams
P3	Male	Allergic eczematous contact dermatitis of local localization, limited to the area where medicated ointments and/or creams were applied	Various medicated ointments and/or creams
P4	Male	Chronic (over 19 years), lichenoid eczema on the skin of the hands, caused by daily occupational exposure to direct contact with wool during professional work	Wool

**Table 7:** Test substances used in the Sulzberger and Lazar experiment [6].

No.	Substance Used in the Experiment	Composition
1	Lanolin fatty acids (Botany Mills, Inc., Passaic, NJ, USA)	1% solution in carbontetrachloride
2	Mixed lanolin alcohols (Botany Mills, Inc., Passaic, NJ, USA)	1% solution in carbontetrachloride
3	Pure lanosterol (Botany Mills, Inc., Passaic, NJ, USA)	1% solution in carbontetrachloride
4	Lanolin fatty acids plus mixed lanolin alcohols (0.5% of each)	1% solution in carbontetrachloride
5	Carbon tetrachloride	as is
6	Ointment base (a commonly used proprietary ointment base, probably containing lanolin alcohols, described as “a mixture of liquid and solid aliphatic hydrocarbons and alcohols obtained by saponification of wool fat”)	as is
7	Lanolin A (Botany Mills, Inc., Passaic, NJ, USA)	as is
8	Lanolin B (New York Pharmacy, New York, NY, USA)	as is
9	Lanolin C (New York Pharmacy, New York, NY, USA)	as is
10	Cholesterol B (Botany Mills, Inc., Passaic, NJ, USA)	1% solution in carbontetrachloride
11	Lanosterol (Botany Mills, Inc., Passaic, NJ, USA)	1% solution in carbontetrachloride
12	Cholesterol A (derived from the spinal cord of cattle)	1% solution in carbontetrachloride

**Table 8:** Results of the Sulzberger and Lazar experiment [6].

No.	Tested Substances	Patients (P)				Controls (C)		
		P1	P2	P3	P4	C1	C2	C3
1	Lanolin fatty acids	0	0	0	0	0	0	0
2	Mixed lanolin alcohols	2(+)/3(+)	1(+)/2(+)	2(+)	1(+)/2(+)	0	0	0
3	Pure lanosterol	0	0	0	0	0	0	0
4	Lanolin fatty acids plus mixed lanolin alcohols	2(+)	2(+)	2(+)	1(+)/2(+)	0	0	0
5	Carbon tetrachloride	0	0	0	0	0	0	0
6	Ointment base	2(+)/3(+)	2(+)	2(+)/3(+)	1(+)/2(+)	0	0	0
7	Lanolin A	1(+)	3(+)	2(+)	1(+)	0	0	0
8	Lanolin B	1(+)	3(+)	2(+)	1(+)/2(+)	0	0	0
9	Lanolin C	2(+)/3(+)	3(+)	2(+)	1(+)/2(+)	0	0	0
10	Cholesterol B	0	0	0	0	0	0	0
11	Lanosterol	0	0	0	0	0	0	0
12	Cholesterol A	0	0	0	0	0	0	0

Legend: 0—no reaction; 1(+)—erythema; 2(+)—erythema and edema; 3(+)—erythema, edema, and vesiculation.

**Table 9:** “Lanolin paradoxes” according to R. Wolf [112].

Lanolin Paradox (No.)	Characteristic Features
No. 1	Medicines containing lanolin may sensitize patients, especially those suffering from atopic dermatitis, while the same lanolin that is present in various cosmetics, commonly used by millions of people, does not cause any adverse reactions in them
No. 2	Medicines containing lanolin may cause allergic contact dermatitis when applied to ulcerated skin, but cosmetics containing lanolin that are applied to healthy skin in the same patients do not cause any irritation (this paradox is especially common in patients with leg ulcers)
No. 3	People sensitive to lanolin often show false negative reactions in patch tests to pure lanolin
No. 4	The use of only 30% lanolin alcohols in patch tests to diagnose hypersensitivity to lanolin is insufficient to detect this allergy and may cause both false negative and false positive results (this paradox is related to the third paradox)

**Table 10:** Combinations of positive results in the retrospective study of Matthieu and Dockx [61].

Patch Test Substance	Positive Reactions in Patch Tests	
	Number of Positive Results/Number of Patients	Percentage of Positive Results
Lanolin alcohols (30%) and/or Amerchol L-101 (100%)	45/393	11.5%
Lanolin alcohols (30%)—only	1/393	0.3%
Amerchol L-101 (100%)—only	32/393	8.1%
Lanolin alcohols (30%) and/or Amerchol L-101 (100%) and/or Amerchol L-101 (50%)	28/223	12.6%
Amerchol L-101 (100%) and Amerchol L-101 (50%)	5/223	2.2%
Amerchol L-101 (50%)—only	8/223	3.6%
Lanolin alcohols (30%) and/or Amerchol L-101 (50%)	0/223	0%

## Data Availability

Not applicable.

## References

[1] Lee B., Warshaw E. (2008). Lanolin allergy: History, epidemiology, responsible allergens, and management. Dermatitis.

[2] Liebreich O. (1890). Note on the Employment of Impure Lanolin (Oesypus) as an External Application in Classical Times. Glasg. Med. J..

[3] https://patentimages.storage.googleapis.com/76/06/df/f797626d43e356/US271192.pdf.

[4] Johnson H., Norman T., Adler B.L., Yu J. (2023). Lanolin: The 2023 American Contact Dermatitis Society Allergen of the Year. Cutis.

[5] Marcus A. (1922). Zum Kapital der Hautkrankheiten auf “nervöser” Basis. Munch. Med. Wochenschr..

[6] Sulzberger M.B., Lazar M.P. (1950). A study of the allergenic constituents of lanolin (wool fat). J. Investig. Dermatol..

[7] Sulzberger M.B., Warshaw T., Herrmann F. (1953). Studies of skin-hypersensitivity to lanolin. J. Investig. Dermatol..

[8] Schlossman M.L., McCarthy J.P. (1978). Lanolin and its derivatives. J. Am. Oil. Chem. Soc..

[9] Tinto W.F., Elufioye T.O., Roach J., Badal S., Delgoda R. (2017). Chapter 22—Waxes. Pharmacognosy.

[10] Chapter: Pharmacognosy and Phytochemistry: Drugs Containing Lipids. https://www.pharmacy180.com/article/lanolin-306/.

[11] Sengupta A., Behera J. (2014). Comprehensive view on chemistry, manufacturing and applications of lanolin extracted from wool pretreatment. Am. J. Eng. Res..

[12] Safety Assessment of Lanolin-Derived Ingredients as Used in Cosmetics. https://www.cir-safety.org/sites/default/files/Lanolin.pdf.

[13] Chemtob C., Fawaz F., Puiseux F. (1974). Analysis of ointments, oils and waxes. XVIII. Study of the chemical composition of liquid lanolin. II. Study of alcohols. Ann. Pharm. Fr..

[14] https://www.cir-safety.org/sites/default/files/TAR_Lanolin_032024.pdf.

[15] Alonso C., Collini I., Martí M., Barba C., Coderch L. (2021). Lanolin-Based Synthetic Membranes for Transdermal Permeation and Penetration Drug Delivery Assays. Membranes.

[16] Carrer V., Guzmán B., Martí M., Alonso C., Coderch L. (2018). Lanolin-Based Synthetic Membranes as Percutaneous Absorption Models for Transdermal Drug Delivery. Pharmaceuticals.

[17] Chrzastek L., Dondela B., Deska M. (2015). Safe ingredients of cosmetics—Lipids and their derivatives. Int. J. Eng. Saf. Sci..

[18] Clark E.W. (1992). Short-term penetration of lanolin into human stratum corneum. J. Soc. Cosmet. Chem..

[19] Clark E.W., Steel I. (1993). Investigations into biomechanisms of the moisturizing function of lanolin. J. Soc. Cosmet. Chem..

[20] Pavlou P., Siamidi A., Varvaresou A., Vlachou M. (2021). Skin Care Formulations and Lipid Carriers as Skin Moisturizing Agents. Cosmetics.

[21] Dixit S. (2001). Lanolin for Silky, Soft, Smooth, Skin. Chem. Weekly.

[22] https://www.nivea.co.uk/about-us/nivea-history.

[23] Jacob S. (2014). The lanolion-woll wax alcohol update. Consultant.

[24] Yao L., Hammond E.G. (2006). Isolation and melting properties of branched-chain esters from lanolin. J. Am. Oil. Chem. Soc..

[25] Płocica J., Tal-Figiel B., Figiel W. (2013). Correlation between rheological studies and organoleptic cosmetic emulsion with lanolin—Natural emulsifier. Tech. Trans.—Chem..

[26] Uter W., Schnuch A., Geier J. (2018). Contact sensitization to lanolin alcohols and Amerchol^®^L101—Analysis of IVDK data. Contact Dermat..

[27] Knijp J., Bruynzeel D.P., Rustemeyer T. (2019). Diagnosing lanolin contact allergy with lanolin alcohol and Amerchol L101. Contact Dermat..

[28] Sulzberger M.B., Morse J.L. (1931). Hypersensitivity to Wool Fat-Report of 2 Cases. JAMA.

[29] Sezary A. (1936). Intolerance cutanée a la lanolin. Presse Med..

[30] Bonnevie P. (1939). Aetiologie und Pathogenese der Ekzemkrankheiten: Klinische Studien über die Ursachen der Ekzeme unter besonderer Berücksichtigung des diagnostischen Wertes der Ekzemproben. Arch. Derm. Syphilol..

[31] Ellis F.A. (1947). Allergic contact dermatitis due to wool fat and cholesterol. Arch. Derm. Syphilol..

[32] Thune P. (1969). Allergy to wool fat. The addition of salicylic acid for patch test purposes. Acta Derm. Venereol..

[33] Silverberg J.I., Patel N., Warshaw E.M., DeKoven J.G., Atwater A.R., Belsito D.V., Dunnick C.A., Houle M.C., Reeder M.J., Maibach H.I. (2022). Lanolin Allergic Reactions: North American Contact Dermatitis Group Experience, 2001 to 2018. Dermatitis.

[34] Jenkins B.A., Belsito D.V. (2023). Lanolin. Dermatitis.

[35] https://pubmed.ncbi.nlm.nih.gov/?term=lanolin+allergy&sort=pubdate&size=200.

[36] https://pubmed.ncbi.nlm.nih.gov/?term=lanolin+hypersensitivity&sort=pubdate&size=200.

[37] Warshaw T.G. (1953). On the Incidcnce of Allergic Skin Reactions to Lanolin, to its Components and Certain Lanolin Modification. J. Soc. Cosmet. Chem..

[38] Baer R.L., Serri F., Weissenbachvial C. (1955). Studies on allergic sensitization to certain topical therapeutic agents. AMA Arch. Derm..

[39] Hjorth N. (1959). Cosmetic allergy. J. Soc. Cosmet. Chem..

[40] Bandmann H.J., Reichenberger M. (1957). Beobachtungen und Untersuchungen zur Frage der durch Eucerin bedingten seltenen Allergie. Der Hautarzt..

[41] Hjorth N., Trolle-Lassen C. (1963). Skin reactions to ointment bases. Trans. St. Johns. Hosp. Dermatol. Soc..

[42] Wereide K. (1965). Contact allergy to wool-fat (“lanolin”). Its incidence in a dermatological in-patient department. Acta Derm. Venereol..

[43] Reichenberger M. (1965). Zur epicutanen Sensibilisierung bei Ulcus cruris-Kranken. Über gehäufte Eucerin allergie. Arch. Klin. Exp. Derm..

[44] Vollum D.I. (1969). Sensitivity to hydrogenated lanolin. Arch. Dermatol..

[45] Epstein E. (1972). The detection of lanolin allergy. Arch. Dermatol..

[46] Angelini G., Rantuccio F., Meneghini C.L. (1975). Contact dermatitis in patients with leg ulcers. Contact Dermat..

[47] Sugai T., Higashi J. (1975). Hypersensitivity to hydrogenated lanolin. Contact Dermat..

[48] Hannuksela M., Kousa M., Pirilä V. (1976). Allergy to ingredients of vehicles. Contact Dermat..

[49] Mortensen T. (1979). Allergy to lanolin. Contact Dermat..

[50] von Liebe V., Karge H.J., Burg G. (1979). Contac turticaria. Hautarzt.

[51] Jenni C., Zala L. (1980). Eczema of the lower leg—Clinical, allergological and differential diagnostic aspects. Schweiz. Med. Wochenschr..

[52] Förg T., Burg G., Zirbs S. (1982). Frequency of contact allergy in housewives. Derm. Beruf. Umwelt..

[53] Frenzel U., Gutekunst A. (1985). Allergic phenomena in the treatment of leg ulcer. Phlebologie.

[54] Edman B. (1985). Sites of contact dermatitis in relationship to particular allergens. Contact Dermat..

[55] Wilson C.L., Cameron J., Powell S.M., Cherry G., Ryan T.J. (1991). High incidence of contact dermatitis in leg-ulcer patients--implications for management. Clin. Exp. Dermatol..

[56] Lever R., Forsyth A. (1992). Allergic contact dermatitis in atopic dermatitis. Acta Derm. Venereol. Suppl..

[57] Pasche-Koo F., Piletta P.A., Hunziker N., Hauser C. (1994). High sensitization rate to emulsifiers in patients with chronic leg ulcers. Contact Dermat..

[58] Dotterud L.K., Falk E.S. (1995). Contact allergy in relation to hand eczema and atopic diseases in north Norwegian schoolchildren. Acta Paediatr..

[59] Van Ginkel C.J., Bruintjes T.D., Huizing E.H. (1995). Allergy due to topical medications in chronic otitis externa and chronic otitis media. Clin. Otolaryngol. Allied Sci..

[60] Schauder S., Ippen H. (1997). Contact and photocontact sensitivity to sunscreens. Review of a 15-year experience and of the literature. Contact Dermat..

[61] Matthieu L., Dockx P. (1997). Discrepancy in patch test results with wool wax alcohols and Amerchol L-101. Contact Dermat..

[62] Le Coz C.J., Scrivener Y., Santinelli F., Heid E. (1998). Sensibilisation de contact au cours des ulcères de jambe (Contact sensitization in leg ulcers). Ann. Dermatol. Venereol..

[63] Reichert-Pénétrat S., Barbaud A., Weber M., Schmutz J.L. (1999). Leg ulcers. Allergologicstudies of 359 cases. Ann. Dermatol. Venereol..

[64] Giordano-Labadie F., Rancé F., Pellegrin F., Bazex J., Dutau G., Schwarze H.P. (1999). Frequency of contact allergy in children with atopic dermatitis: Results of a prospective study of 137 cases. Contact Dermat..

[65] Wakelin S.H., Smith H., White I.R., Rycroft R.J., McFadden J.P. (2001). A retrospective analysis of contact allergy to lanolin. Br. J. Dermatol..

[66] Machet L., Couhé C., Perrinaud A., Hoarau C., Lorette G., Vaillant L. (2004). A high prevalence of sensitization still persists in leg ulcer patients: A retrospective series of 106 patients tested between 2001 and 2002 and a meta-analysis of 1975–2003 data. Br. J. Dermatol..

[67] Deleo V.A., Taylor S.C., Belsito D.V., Fowler J.F., Fransway A.F., Maibach H.I., Marks J.G., Mathias C.G., Nethercott J.R., Pratt M.D. (2002). The effect of race and ethnicity on patch test results. J. Am. Acad. Dermatol..

[68] Kieć-Świerczyńska M., Kręcisz B., Świerczyńska-Machura D. (2003). Most frequent causes of allergic contact dermatitis in farmers: Based on material in the Nofer Institute of Occupational Medicine, Lodz. Med. Pr..

[69] Machovcova A., Dastychova E., Kostalova D., Vojtechovska A., Reslova J., Smejkalova D., Vaneckova J., Vocilkova A. (2005). Common contact sensitizers in the Czech Republic. Patch test results in 12,058 patients with suspected contact dermatitis. Contact Dermat..

[70] Goon A.T., Goh C.L. (2006). Patch testing of Singapore children and adolescents: Our experience over 18 years. Pediatr. Dermatol..

[71] Oppel T., Schnuch A. (2006). The most frequent allergens in allergic contact dermatitis. Dtsch. Med. Wochenschr..

[72] Tomljanović-Veselski M., Lipozencić J., Lugović L. (2007). Contact allergy to special and standard allergens in patients with venous ulcers. Coll. Antropol..

[73] Beattie P.E., Green C., Lowe G., Lewis-Jones M.S. (2007). Which children should we patch test?. Clin. Exp. Dermatol..

[74] Smart V., Alavi A., Coutts P., Fierheller M., Coelho S., Linn Holness D., Sibbald R.G. (2008). Contact allergens in persons with leg ulcers: A Canadian study in contact sensitization. Int. J. Low. Extrem. Wounds.

[75] Hogeling M., Pratt M. (2008). Allergic contact dermatitis in children: The Ottawa hospital patch-testing clinic experience, 1996 to 2006. Dermatitis.

[76] Warshaw E.M., Nelsen D.D., Maibach H.I., Marks J.G., Zug K.A., Taylor J.S., Rietschel R.L., Fowler J.F., Mathias C.G., Pratt M.D. (2009). Positive patch test reactions to lanolin: Cross-sectional data from the north american contact dermatitis group, 1994 to 2006. Dermatitis.

[77] Schnuch A., Szliska C., Uter W. (2009). Facial allergic contact dermatitis. Data from the IVDK and review of literature. Hautarzt.

[78] Nguyen J.C., Chesnut G., James W.D., Saruk M. (2010). Allergic contact dermatitis caused by lanolin (wool) alcohol contained in an emollient in three postsurgical patients. J. Am. Acad. Dermatol..

[79] Minamoto K. (2010). Skin sensitizers in cosmetics and skin care products. Nihon Eiseigaku Zasshi. Jpn. J. Hyg..

[80] Beliauskienė A., Valiukevičienė S., Sitkauskienė B., Schnuch A., Uter W. (2011). Contact sensitization to the allergens of European baseline series in patients with chronic leg ulcers. Medicina.

[81] Fellinger C., Hemmer W., Wantke F., Wöhrl S., Jarisch R. (2013). Severe allergic dermatitis caused by lanolin alcohol as part of an ointment base in propolis cream. Contact Dermat..

[82] Miest R.Y., Yiannias J.A., Chang Y.H., Singh N. (2013). Diagnosis and prevalence of lanolin allergy. Dermatitis.

[83] Fraser K., Pratt M. (2015). Polysensitization in recurrent lip dermatitis. J. Cutan. Med. Surg..

[84] Warshaw E.M., Maibach H.I., Taylor J.S., Sasseville D., DeKoven J.G., Zirwas M.J., Fransway A.F., Mathias C.G., Zug K.A., DeLeo V.A. (2015). North American contact dermatitis group patch test results: 2011–2012. Dermatitis.

[85] Belloni Fortina A., Cooper S.M., Śpiewak R., Fontana E., Schnuch A., Uter W. (2015). Patch test results in children and adolescents across Europe. Analysis of the ESSCA Network 2002–2010. Pediatr. Allergy Immunol..

[86] Mahler V. (2015). Contact allergies in the elderly. Hautarzt.

[87] Higgins C.L., Nixon R.L. (2016). Periorbital Allergic Contact Dermatitis Caused by Lanolin in a Lubricating Eye Ointment. Australas. J. Dermatol..

[88] Uter W., Śpiewak R., Cooper S.M., Wilkinson M., Sánchez Pérez J., Schnuch A., Schuttelaar M.L. (2016). Contact allergy to ingredients of topical medications: Results of the European Surveillance System on Contact Allergies (ESSCA), 2009–2012. Pharmacoepidemiol. Drug. Saf..

[89] Erfurt-Berge C., Geier J., Mahler V. (2017). The current spectrum of contact sensitization in patients with chronic leg ulcers or stasis dermatitis - new data from the Information Network of Departments of Dermatology (IVDK). Contact Dermat..

[90] Lubbes S., Rustemeyer T., Sillevis Smitt J.H., Schuttelaar M.L., Middelkamp-Hup M.A. (2017). Contact sensitization in Dutch children and adolescents with and without atopic dermatitis - a retrospective analysis. Contact Dermat..

[91] Jacob S.E., McGowan M., Silverberg N.B., Pelletier J.L., Fonacier L., Mousdicas N., Powell D., Scheman A., Goldenberg A. (2017). Pediatric Contact Dermatitis Registry Data on Contact Allergy in Children with Atopic Dermatitis. J. Am. Acad. Dermatol..

[92] Fransen M., Overgaard L.E.K., Johansen J.D., Thyssen J.P. (2018). Contact allergy to lanolin: Temporal changes in prevalence and association with atopic dermatitis. Contact Dermat..

[93] Pap E.B., Temesvári E., Németh I., Sárdy M., Pónyai G. (2018). Contact hypersensitivity in adolescents. Pediatr. Dermatol..

[94] Rastogi S., Patel K.R., Singam V., Silverberg J.I. (2018). Allergic contact dermatitis to personal care products and topical medications in adults with atopic dermatitis. J. Am. Acad. Dermatol..

[95] Németh D., Pónyai G. (2022). Contact Allergy in the Elderly: A Study of 600 Patients. Life.

[96] Németh D., Temesvári E., Holló P., Pónyai G. (2022). Preservative Contact Hypersensitivity among Adult Atopic Dermatitis Patients. Life.

[97] Everall J., Truter E.V. (1954). Cutaneous hypersensitivity to lanolin; investigation of one case. J. Investig. Dermatol..

[98] Clark E.W., Cronin E., Wilkinson D.S. (1977). Lanolin with reduced sensitizing potential. A preliminary note. Contact Dermat..

[99] Clark E.W., Blondeel A., Cronin E., Oleffe J.A., Wilkinson D.S. (1981). Lanolin of reduced sensitizing potential. Contact Dermat..

[100] Bourrinet P., Berkovic A. (1980). Cutaneous hypersensitivity tests in guinea pig of lanolin and derivatives. Ann. Pharm. Fr..

[101] Bourrinet P., Berkovic A. (1981). Tests of skin hypersensitivity of guinea pigs to lanolin and derivatives used in cosmetology. Int. J. Cosmet. Sci..

[102] Fregert S., Dahlquist I., Trulsson L. (1984). An attempt to isolate and identify allergens in lanolin. Contact Dermat..

[103] Oleffe L.A., Blondeel A., Boschmans S. (1978). Patchtesting with lanolin. Contact Dermat..

[104] Kligman A.M. (1983). Lanolin allergy: Crisis or comedy. Contact Dermat..

[105] Kligman A.M. (1998). The myth of lanolin allergy. Contact Dermat..

[106] Schwarzfeld H.K. (1952). Sensitivity to ointments containing wool fat. U.S. Armed Forces Med. J..

[107] Pérez A., González G., González J., Heinzen H. (2010). Multiresidue Determination of Pesticides in Lanolin Using Matrix Solid-Phase Dispersion. J. AOAC Int..

[108] Nurse D.S. (1987). Dangers of the application of lanolin. Med. J. Aust..

[109] Morse J. (1989). The hazards of lanolin. MCN Am. J. Matern. Child. Nurs..

[110] Paton M.W., Petterson D.S. (1997). Absorption by sheep of dieldrin from contaminated soil. Aust. Vet. J..

[111] Rosanove R. (1987). Dangers of the application of lanolin. Med. J. Aust..

[112] Wolf R. (1996). The lanolin paradox. Dermatology.

[113] Fisher A.A. (1973). The paraben paradox. Cutis.

[114] Geier J., Uter W., Pirker C., Frosch P.J. (2003). Patch testing with the irritant sodium lauryl sulfate (SLS) is useful in interpreting weak reactions to contact allergens as allergic or irritant. Contact Dermat..

[115] Uter W., Hegewald J., Pfahlberg A., Pirker C., Frosch P.J., Gefeller O. (2003). The association between ambient air conditions (temperature and absolute humidity), irritant sodium lauryl sulfate patch test reactions and patch test reactivity to standard allergens. Contact Dermat..

[116] Brasch J., Schnuch A., Uter W. (2003). Patch-test reaction patterns in patients with a predisposition to atopic dermatitis. Contact Dermat..

[117] Zallmann M., Smith P.K., Tang M.L.K., Spelman L.J., Cahill J.L., Wortmann G., Katelaris C.H., Allen K.J., Su J.C. (2017). Debunking the Myth of Wool Allergy: Reviewing the Evidence for Immune and Non-immune Cutaneous Reactions. Acta Derm. Venereol..

[118] Uldahl A., Engfeldt M., Svedman C. (2021). Clinical relevance of positive patch test reactions to lanolin: A ROAT study. Contact Dermat..

[119] Bourke J.F., Batta K., Prais L., Abdullah A., Foulds I.S. (1999). The reproducibility of patch tests. Br. J. Dermatol..

[120] Ale S.I., Maibach H.I. (2004). Reproducibility of patch test results: A concurrent right-versus-left study using TRUE Test. Contact Dermat..

[121] Adya K.A., Inamadar A.C., Palit A. (2013). Paradoxes in dermatology. Indian Dermatol. Online J..

[122] Türsen Ü. (2019). Paradoxes in Aesthetic Dermatology. J. Turk. Acad. Dermatol..

